# Section on Pathology, February 18, 1902

**Published:** 1902-04

**Authors:** Irving P. Lyon

**Affiliations:** Secretary


*Section on Pathology, February 18, 1902.*

Reported by IRVING P. LYON, M. D., Secretary.

The regular monthly meeting of the section of pathology of the Buffalo Academy of Medicine, was held in the Academy rooms, Public Library Building, February 18, 1902, at 8.30 p.m., Dr. Grover W. Wende, chairman, presiding.

The essayist was Dr. John Addison Fordyce, of New York, whose subject was

CLINICAL AND PATHOLOGICAL CASES IN DERMATOLOGY, ILLUSTRATED BY STEREOPTICON.

The numerous photographs and photomicrographs presented on the projection screen were remarkable for their clearness and the wide range of dermatology and pathology embraced by them, being the personal work of Dr. Fordyce, preëminent in this field of work. These numerous illustrations were entertainingly discussed in an informal style by Dr. Fordyce.

Dr. Leo Loeb, of Chicago, presented specimens of white rats experimentally inoculated with sarcoma by injection into the peritoneal cavity of the filtered (through filter paper) juices of sarcoma. The original sarcoma was found in the thyroid gland of a white rat. The experimental tumors conformed always to the histological type of the original tumor. Metastases were obtained in some of the experimental white rats. Such an example was exhibited, a white rat showing the abdominal cavity filled with sarcomatous masses.

In discussing the subject, Dr. HARVEY R. GAYLORD stated that Dr. Loeb had succeeded in carrying the inoculation of sarcoma through a series of at least several hundred white rats.

Dr. A. L. BENEDICT inquired whether Dr. Loeb's experiment threw any light on the question of the possible identity of sarcoma and carcinoma, the histological difference depending upon the localisation of the process respectively in mesoblastic or in epithelial tissues. Dr. Loeb replied that he had never obtained carcinoma in his experimental animals. Moreover, the sarcoma produced always conformed to the type of the original tumor.

Dr. C. E. CONGDON asked whether the experimental tumors had been obtained in animals of different species from that of the animal showing the original tumor. Dr. Loeb replied that the original tumor was from a white rat and gave positive results only when transplanted into white rats or hybrids from white rats and common house rats. Negative results were always obtained in attempts to transplant the growths into chickens, guinea-pigs, white mice, rabbits and the like.

Dr. IRVING P. LYON remarked the apparent specificity of the process, requiring, as it did, that the tumor be transplanted into the identical species of animal (white rat) from which it originally came, or at the farthest, a hybrid produced by crossing the original species with a nearly related species (*i. e.*, white rat and common house rat). This fact indicated one of two things: either a specificity of an infecting microörganism or a specificity of the infecting pathologic cell, or, perhaps, a kind of combination of both—namely, a specificity of the pathologic cell itself infected with a microörganism. Hence the negative results obtained from the attempt to experimentally reproduce cancer in the lower animals from human cancer. The hope of solving the cancer question, therefore, seemed to lie in working upon animal cancer and inoculating this cancer in animals of the same species, as Dr. Loeb had done. Inoculation direct from human beings to animals was probably impossible.

Members present, 57. Adjournment, 10.40 p.m.

